# Mining geriatric assessment data for in-patient fall prediction models and high-risk subgroups

**DOI:** 10.1186/1472-6947-12-19

**Published:** 2012-03-14

**Authors:** Michael Marschollek, Mehmet Gövercin, Stefan Rust, Matthias Gietzelt, Mareike Schulze, Klaus-Hendrik Wolf, Elisabeth Steinhagen-Thiessen

**Affiliations:** 1 Peter L. Reichertz Institute for Medical Informatics, University of Braunschweig - Institute of Technology and Hanover Medical School, Carl-Neuberg-Str. 1, 30625 Hanover, Germany; 2 Peter L. Reichertz Institute for Medical Informatics, University of Braunschweig - Institute of Technology and Hanover Medical School, Mühlenpfordtstr. 23, 38106 Braunschweig, Germany; 3 Geriatrics Research Group, Department of Geriatric Medicine, Charité University Medicine, Reinickendorfer Str. 61, 13347 Berlin, Germany

**Keywords:** Accidental falls, Geriatric assessment, Data mining

## Abstract

**Background:**

Hospital in-patient falls constitute a prominent problem in terms of costs and consequences. Geriatric institutions are most often affected, and common screening tools cannot predict in-patient falls consistently. Our objectives are to derive comprehensible fall risk classification models from a large data set of geriatric in-patients' assessment data and to evaluate their predictive performance (aim#1), and to identify high-risk subgroups from the data (aim#2).

**Methods:**

A data set of n = 5,176 single in-patient episodes covering 1.5 years of admissions to a geriatric hospital were extracted from the hospital's data base and matched with fall incident reports (n = 493). A classification tree model was induced using the C4.5 algorithm as well as a logistic regression model, and their predictive performance was evaluated. Furthermore, high-risk subgroups were identified from extracted classification rules with a support of more than 100 instances.

**Results:**

The classification tree model showed an overall classification accuracy of 66%, with a sensitivity of 55.4%, a specificity of 67.1%, positive and negative predictive values of 15% resp. 93.5%. Five high-risk groups were identified, defined by high age, low Barthel index, cognitive impairment, multi-medication and co-morbidity.

**Conclusions:**

Our results show that a little more than half of the fallers may be identified correctly by our model, but the positive predictive value is too low to be applicable. Non-fallers, on the other hand, may be sorted out with the model quite well. The high-risk subgroups and the risk factors identified (age, low ADL score, cognitive impairment, institutionalization, polypharmacy and co-morbidity) reflect domain knowledge and may be used to screen certain subgroups of patients with a high risk of falling. Classification models derived from a large data set using data mining methods can compete with current dedicated fall risk screening tools, yet lack diagnostic precision. High-risk subgroups may be identified automatically from existing geriatric assessment data, especially when combined with domain knowledge in a hybrid classification model. Further work is necessary to validate our approach in a controlled prospective setting.

## Background

Falls and their consequences are a well-known and urgent problem in our ageing population. It is known that geriatric in-patients exhibit the highest fall incidence among institutionalized persons, ranging from 6.3 to 7.2% within a period of two weeks [1]. About 20-30% of falls result in injuries that need medical intervention [1,2], among which 3-5% are fractures [2,3]. Apart from the personal consequences of fall events, such as injuries leading to lasting disability and loss of independence or psychological effects such as the post-fall-syndrome [4], they also have economical implications for the health system in general, and for hospitals in particular. The annual costs of falls in the U.S. have been estimated at 19.2$ billion [5].

In consequence, many assessment tools and risk scales have been developed in order to identify in-patients with a potential fall risk, with the aim to apply timely targeted preventive measures to avoid these events in the first place. Gates reports on 29 different assessment tools, among these e.g. the widely-used *Performance-Oriented Mobility Assessment *(POMA) by Tinetti [6], and concludes that no explicit recommendation may be given for any single test or scale [7]. Oliver et al. - authors of the *St. Thomas Risk Assessment Tool in Falling Elderly In-patients *(STRATIFY) [8] - systematically review prospective studies with different assessment scales and conclude that none of these is able to identify a high percentage of fallers correctly [9]. Similar results have been reported by Kim et al. [10]. Most of the available fall risk assessment scales are based on experiential knowledge. The wide-spread use of electronic documentation systems makes large amounts of patient data available. This data can be used to extract information automatically, employing methods of machine learning and data mining, e.g. to generate classification models or to identify specific subgroups of patients who have a high mortality risk [11].

The aim of our research work for this paper is to employ a data mining approach in order

• To derive comprehensible fall risk classification models from a large data set of geriatric in-patients' assessment data and to evaluate their predictive performance (aim#1), and

• To identify subgroups within a geriatric in-patient population who have a high fall risk (aim#2).

## Methods

### Study data set

The study data set comprises all in-patient episodes from July 2006 to December 2007 at the *Evangelisches Geriatriezentrum Berlin gGmbH (EGZB)*, being the department for geriatric medicine of the Charité university hospital in Berlin and the largest geriatric clinic in Germany. Altogether n = 5,176 single episodes were extracted from the clinical information system (3,384 female, 1,792 male, *RehaDoc *system). These were matched with the clinic's paper-based fall incident reports, which are filled in for *every *fall event, amounting to n = 493 within the study period. A fall is defined as an unexpected event during which a patient involuntarily comes to rest on the ground. This does not include events during which patients are lowered to the ground by staff members. The average age of the patients was 77.5 years, and their mean Barthel index score was 44.7 points (SD = 26.3 points).

The research for this paper has been conducted in compliance with the Helsinki Declaration. The use of anonymized patient data for research has been approved by the ethics committee of the Charité university hospital, and a consent form to that effect is signed by every patient or her or his legal representative on admission.

As is common in clinical data sets, not all items are available for every patient. This is of course partly due to forgotten entries, but primarily to the fact that several assessment tests or sub-tests have not been performed with the patients, e.g. because they were physically not capable (e.g. for the Performance-Oriented Mobility Assessment [6]), lived in a nursing home (social status questionnaire) or were mentally so inconspicuous that they were not tested at all (*Mini-Mental State Examination*). The items included in the extracted data sets are shown in Table 1 along with the percentage of missing values.

**Table 1:** Items and item sets used to induce the classification models along with the percentages of missing values in our data set (n = 5,176 cases); the 19 Lachs and 22 Tinetti sub-scores are not listed separately

Item (set) name	Missing values in%
Age on admission	0.0

Sex (m/f)	0.0

Social status (35 sub-items concerning social contacts, activities, living, economic situation)	54.3

Barthel index sum score [12]	2.2

Lachs score (16 sub-items [13])	0.0-19.4

Timed 'Up & Go' test total time	58.8

Performance-Oriented Mobility Assessment (POMA) by Tinetti (22 sub-items [6])	31.7-68.5

Mini-Mental State Examination (MMSE) score on admission	53.2

Number of diagnoses on admission	0.7

Number of different medications on admission	1.0

Fall (yes/no)	0.0

All of the above- mentioned items were used for the data mining algorithms, yet only a subset of them actually appears in the models, due to inherent attribute selection processes of the employed algorithms. These are e.g. based on their ability to part subgroups of fallers and non-fallers using the *information gain *(C4.5 algorithm) [14].

### Classification model induction and evaluation

We used two supervised machine learning algorithms to induce classification models, the *C4.5 classification tree *algorithm introduced by Quinlan [15] (minimum number of instances per leaf = 20, confidence factor = 0.25) and a logistic regression algorithm (maximum boosting iterations = 500, cross-validation), as implemented in the Waikato Environment for Knowledge Analysis (WEKA, version 3.7.2) [14]. The classification tree on the one hand is comprehensible, as similar rules resp. diagnostic algorithms are well known among clinicians, and it allows for the extraction of explicit classification rules as well as high-risk subgroups within a population [11], both of which can be useful in clinical practice [16]. Logistic regression models on the other hand are known to be more stable than decision trees with regard to missing data and small changes in the data sets which often lead to changes in tree structure. For both algorithms the binary attribute *fall *(yes/no) was used as reference for the induction of the model. Missing values are treated by the two algorithms using different strategies: C4.5 splits the training data instances to the leaves of the decision tree proportionally to the occurrence of missing data in the data set. The logistic regression algorithm, in contrast, replaces missing values using the means of non-missing values in the training data set.

In order to optimize the models' predictive performance to serve as a screening test, a 2 × 2 cost matrix was employed, defining the relative cost of false negatives - patients who fall but are not identified by the model - as 20-fold higher than those of false positives. This estimate is based on the consideration that false negatives are extremely costly. Despite several published studies on the overall cost of falls (e.g. [5,17]), only few studies exist which compare *long-term costs *of fallers and non-fallers. A recent study by Carroll et al. reports a difference of about 6200US$ per person per year between those two groups [18]. Apart from the cost matrix, a classifier optimization algorithm called *Threshold Selector *[14] was used. It optimizes the *F-measure*, i.e. an established classification performance measure defined as

$$\frac{2\;*\;TP}{2\;*\;TP\;+\;FP\;+\;FN}$$

(TP = true positives, FP = false positives, FN = false negatives)

The evaluation of the classification models was done by means of a ten times ten-fold cross-validation, and performance was assessed by calculating sensitivity, specificity, positive and negative predictive values (PPV/NPV), classification accuracy, the area under the curve (AUC) and the likelihood ratios of positive (+LR) and negative test results (-LR) along with their 95% confidence intervals.

### Risk group identification

In order to identify subgroups with a high in-patient fall risk, classification rules having the condition *fall = yes *as the rule's consequent were read from the decision tree model. In this process, we only considered rules that were applicable to at least 100 instances from our data set - thus having an acceptable coverage -and which had a relative accuracy of at least 70%.

## Results

Table 2 shows the classification results of our decision tree model along with the contingency table. The decision tree itself is not included due to it size of 107 nodes. Following the rules of Good Scientific Practice (e.g. [19]), the complete classification tree may be requested from the first author. The results show that about two thirds (67.1%) of non-fallers, but little more than half (55.4%) of fallers are identified correctly. An NPV of 93.5% is pitted against a low 15% for PPV. The overall classification accuracy amounts to 66%, accompanied by a poor 0.63 AUC value. The likelihood ratios (Table 3) confirm that the results differ from chance, but also that the test results enhance diagnostic accuracy only marginally [20].

**Table 2:** Classification results and contingency table for our decision tree model (n = 5,176)

decision tree model					
				
Classification accuracy	66.0%				
	
sensitivity	55.4%		Prediction		
specificity	67.1%		No	yes	Sum

neg. predictive value	93.5%	no fall	3141	1542	4684

pos. predictive value	15.0%	Fall	220	273	493

AUC	0.63	Sum	3361	1815	5176

**Table 3:** +LR and -LR values of the classification models (decision tree and logistic regression) including their 95% confidence intervals (n = 5,176)

model name	+LR value (95% CI)	- LR value (95% CI)
decision tree	1.68 (1.54-1.84)	0.67 (0.6-0.74)

logistic regression	1.43 (1.32-1.53)	0.66 (0.59-0.74)

Very similar results (Tables 4 and 3) are found by the logistic regression model. While the AUC and the NPV are equal to that of the decision tree, the classification accuracy (56.2%), specificity (55.4%), and PPV (13%) are lower. Only the sensitivity (63.5%) is higher. The + LR and -LR values (Table 3) are similar to that of the decision tree model.

**Table 4:** Classification results and contingency table for our logistic regression model (n = 5,176)

logistic regression model					
				
Classification accuracy	56.2%				
	
sensitivity	63.5%		Prediction		
specificity	55.4%		No	yes	Sum

neg. predictive value	93.5%	no fall	2596	2087	4684

pos. predictive value	13.0%	Fall	180	313	493

AUC	0.63	Sum	2776	2400	5176

Table 5 contains five classification rules representing high risk subgroups, all of which have been extracted with the consequent *fall = yes*, along with their relative accuracy within the data set.

**Table 5:** Classification rules extracted from the decision tree model; only rules with the condition *fall = yes *as consequent and which cover a number of at least 100 instances and have a related accuracy of at least 70% were considered; the rules are ordered by their relative accuracy

**no**.	rule (consequent: *fall=yes*)	relative accuracy
1a b	(Barthel index score ≤ 45 pts) *and *(sex = male) *and *(age > 75y) *and *(Lachs depression item = 0)	84.3% 77.8%

2	(Barthel index score > 10 and ≤ 45 pts) *and *(sex = female) *and *(number of medications < 14) *and *(MMSE score ≤ 26 pts) *and *(institutionalized=yes) *and *(needs aid for standing)	80.0%

3	(Barthel index score > 45 and ≤ 65 pts) *and *(MMSE score > 21 pts) *and *(Timed 'Up& Go' time ≤ 42s) *and *(number of diagnoses > 11) *and *(number of medications > 8)	72.8%

4	(Barthel index score > 45 and ≤ 65 pts) *and *(MMSE score ≤ 18 pts)	72.1%

5	(Barthel index score > 45 and ≤ 65 pts) *and *(MMSE score > 18 pts) *and *(Timed 'Up& Go' time > 42s) *and *(age > 71y)	71.9%

## Discussion

The classification results show that the classification models can only identify slightly more than half (55.4/63.5%) of the patients who will suffer from a fall during their in-patient stay (aim#1). This result is similar to those obtained e.g. by Kim et al. [10] or in the meta analysis performed by Oliver et al. [9], who conclude that even the best tools cannot identify a large majority of fallers. For some of these patients, a fall might be avoided, provided that effective preventive measures are taken in time. This potential benefit, however, is countered by low positive predictive values of just 15/13%, making this approach costly and thus rendering it useless. The negative predictive value, in turn, is high in both models (93.5%), so that patients who will not fall - and therefore do not need specific preventive measures - can be identified correctly. Overall, the results are similar to those obtained in a previous smaller study conducted by some of the authors [21], and they seem disappointing, especially as the test battery contains established and validated tests often used for assessing fall risk, such as the Timed Up & Go [22] or the POMA [6]. On the other hand, a high fall risk is not necessarily associated with an actual fall event which to some extent is random in a short and variable in-patient period of time, even more so if a special environment such as a geriatric ward is the setting. As such, our model very likely suffers from a multitude of influencing factors (e.g. post-operative weakness, unfamiliar environment, problems with sleeping, analgesia medication), part of which are neither assessed during an in-patient stay, nor are controllable.

A closer analysis of the rules defining the high-risk subgroups from the data sets of the 493 fall incidents reveals a number of factors which are associated with a higher than normal risk and are also found in literature as well as are part of experiential clinical knowledge. First of all, an age above 70 years obviously can be regarded as a risk factor, as can a Barthel index score of ≤ 45 pts. The latter reveals a significant limitation in a person's overall ability to cope with daily life, such as toileting or mobility [12]. Old age of course is attributed to frailty, originating e.g. either from sarcopenia or the existence of co-morbidity concerning chronic diseases such as e.g. arthritis or diabetes. Steinhagen-Thiessen and Borchelt e.g. report results from the Berlin Aging Study showing that 88% of the persons aged 70 years or above suffer from at least five somatic diseases [23]. Cognitive impairment as defined by a low MMSE score also constitutes a risk for fall events in geriatric patients [24]. In addition to this, being institutionalized represents a risk, but this result is likely influenced by a negative selection bias, as people are often admitted to institutions because they have become too weak to live independently and for this reason may have an elevated risk [1]. Finally, a high degree of co-morbidity as well as polypharmacy is attributed with a high risk of falling. The latter confirms results reported e.g. by Kojima et al. [25] or Chang et al. [26], and questions asking for certain psychotropic medications are part of e.g. the STRATIFY score [8].

Along with risk group identification, we have to look at therapeutic consequences of identifying potential fallers and predicting in-patient falls. Although we inherently hypothesize that, if we predict these events correctly, we will be able to initiate preventive measures that will avoid at least a certain proportion of fall incidents, we cannot prove this until a sound controlled study has been performed. Also, if viewed from an economic perspective, we currently do not know if the benefit of in-patient fall risk screening and especially the following interventions outweigh the costs of such an endeavor.

## Limitations

Classification trees tend to be unstable in small data sets. Therefore we have also used a logistic regression model in addition. Nevertheless, according to our aim#2 to identify high-risk subgroups, we deliberately chose the decision tree approach [11] despite our medium-sized data set (> 5,000 instances). Furthermore, we expected - and found - non-linear relationships for some parameters (e.g. age), confirming the justification of our choice. The significant amount of missing data for some sub-items limits the generalizability of our findings, yet this is quite normal in clinical data sets where it is often neither necessary nor practical to apply all available test procedures. We have used model induction algorithms that employ two different strategies of dealing with missing data, thus minimizing the effect. Finally, the cost matrix defining the costs of false negatives as being 20-fold higher than those of false positives, is a rough estimate as mentioned above and therefore is to some extent arbitrary, as the authors are not aware of an explicit study providing a ratio comparing long-term in-patient fall-related costs with those of non-fallers.

## Conclusion

Based on more than 5,000 data sets obtained from a clinical data base of a geriatric hospital, we generated two classification models that are able to detect 55.4/63.5% of fallers and 67.1/55.4% of non-fallers correctly. Furthermore, we identified five subgroups with a high risk of falling during an in-patient stay. The description of these groups and the interpretation of the risk factors found *(age, low ADL score, cognitive impairment, institutionalization, polypharmacy and co-morbidity) *may be useful in future practice for screening geriatric patients on admission to a hospital for their individual risk. Our future work will include the generation of a new hybrid risk classification model that incorporates both medical domain knowledge as well as the knowledge gained from our data mining approach. Such a model could be updated repeatedly with new data, enabling its customization for different populations of patients or even different hospital environments. Finally, further research work is needed to evaluate our models in a prospective controlled setting as well as from an economic perspective.

## Competing interests

The authors declare that they have no competing interests.

## Authors' contributions

MM supervised the study, carried out the data analysis and drafted the manuscript. MG organized the data export from the hospital information system and interpreted the results of the analysis. SR preprocessed the data sets and conducted part of the data analysis. MGie provided statistical and methodological feedback for the analysis. MS participated in the data analysis and the computation of performance values. KHW participated in drafting the study protocol. EST conceived of the study and participated in its coordination. All authors read and approved the final manuscript.

## Pre-publication history

The pre-publication history for this paper can be accessed here:

http://www.biomedcentral.com/1472-6947/12/19/prepub
